# Bacterial Degradation of Aromatic Compounds

**DOI:** 10.3390/ijerph6010278

**Published:** 2009-01-13

**Authors:** Jong-Su Seo, Young-Soo Keum, Qing X. Li

**Affiliations:** 1 Department of Molecular Biosciences and Bioengineering, University of Hawaii, 1955 East-West Road, Honolulu, HI 96822, USA; 2 Current address: Analytical Research Center, Korea Institute of Toxicology, 100 Jangdong, Yuseonggu, Daejeon 305–343, Korea; E-Mail: jsseo@kitox.re.kr (J.-S.S.);; 3 Current address: School of Agricultural Biotechnology, Seoul National University, San 56–1, Shinrim-9-dong, Kwanakgu, Seoul 151–742, Korea; E-mail: rational@snu.ac.kr (Y.-S.K.)

**Keywords:** Bioremediation, biodegradation, PAHs, polycyclic aromatic hydrocarbons

## Abstract

Aromatic compounds are among the most prevalent and persistent pollutants in the environment. Petroleum-contaminated soil and sediment commonly contain a mixture of polycyclic aromatic hydrocarbons (PAHs) and heterocyclic aromatics. Aromatics derived from industrial activities often have functional groups such as alkyls, halogens and nitro groups. Biodegradation is a major mechanism of removal of organic pollutants from a contaminated site. This review focuses on bacterial degradation pathways of selected aromatic compounds. Catabolic pathways of naphthalene, fluorene, phenanthrene, fluoranthene, pyrene, and benzo[*a*]pyrene are described in detail. Bacterial catabolism of the heterocycles dibenzofuran, carbazole, dibenzothiophene, and dibenzodioxin is discussed. Bacterial catabolism of alkylated PAHs is summarized, followed by a brief discussion of proteomics and metabolomics as powerful tools for elucidation of biodegradation mechanisms.

## 1. Introduction

Biodegradation is a viable bioremediation technology for organic pollutants. It has long known that microorganisms degrade environmental pollutants in various matrices and environments. Bioremediation utilizes the metabolic versatility of microorganisms to degrade hazardous pollutants. A goal of bioremediation is to transform organic pollutants into harmless metabolites or mineralize the pollutants into carbon dioxide and water [1]. A feasible remedial technology requires microorganisms being capable of quick adaptation to and efficient uses of pollutants of interest in a particular case in a reasonable period of time. Many factors influence microorganisms to use pollutants as substrates or cometabolize them. Therefore, understanding catabolic pathways and mechanisms and responsible enzymes is an effective means to define important factors for efficient cleanup of pollutants. Research has been conducted to understand bioremediation for environmental pollutants such as aromatic compounds that are among the most prevalent and persistent environmental pollutants.

Biodegradation is a very broad field and involves uses of a wide range of microorganisms to break chemical bonds. It has been well reviewed [1, 2], however, it is a very active field and new data are rapidly contributed to the literature. This review is focused on bacterial catabolic pathways of selected aromatic pollutants under aerobic culture conditions (Table 1). The selected aromatic pollutants include the PAHs naphthalene, fluorene, phenanthrene, fluoranthene, pyrene, and benzo[*a*]pyrene, the heterocycles dibenzofuran, carbazole, dibenzothiophene, and dibenzodioxin, and alkylated PAHs. Metabolomics and proteomics in elucidation of mechanisms of microbial degradation of aromatics are also briefly discussed.

## 2. Aromatic Compounds in the Environment

Aromatic compounds can be defined as organic molecules containing one or more aromatic rings, specifically benzene rings, for example. Different aromatic compounds co-exist as complex mixtures in petroleum refinery and distillation sites. There are three major categories: polycyclic aromatic hydrocarbons (PAHs), heterocyclics, and substituted aromatics. PAHs are a group of chemicals that contain two or more fused aromatic rings in linear, angular, or cluster arrangements [3, 4]. Physical and chemical properties of PAHs vary with the number of rings and hence their molecular weight. Chemical reactivity, aqueous solubility and volatility of PAHs decrease with increasing molecular weight. As a result, PAHs differ in their transport, distribution and fate in the environment and their effects on biological systems. The US EPA has identified 16 PAHs as priority pollutants. Some of these PAHs are considered to be possible or probable human carcinogens, and hence their distributions in the environment and possible exposure to humans have been of concerns [5]. High-molecular-weight PAHs are paid particular attention as they are recalcitrant [4]. In general, PAHs are relatively stable and recalcitrant in soils and less easy to degrade than many other organic compounds. They are difficult to remove from contaminated soil using the treatments that have been used successfully to clean soils contaminated with more degradable or volatile organic compounds such as alkanes [6]. Three major sources of PAHs are petrogenic, pyrogenic and biogenic. Petrogenic PAHs are from petroleum and petroleum-derived products, and are often marked as in abundance of alkyl-substituted PAHs such as alkyl naphthalenes, alkyl phenanthrenes and alkyl dibenzothiophenes. Pyrogenic PAHs are produced from combustion processes and are comprised of predominantly unsubstituted PAHs. Biogenic aromatic compounds including aromatic amino acids, lignin compounds and their derivatives are of biotransformation origin.

PAHs may accumulate in high concentrations in terrestrial environments near coal gasification sites and tar oil distillation plants [7]. Major sources of PAHs are incomplete combustion of organic materials, gas production, wood treatment facilities, and waste incineration [3, 8–10]. PAHs are formed naturally during thermal geologic reactions associated with fossil-fuel and mineral production, and during burning of vegetation in forest and bush fires [11, 12]. Anthropogenic sources, particularly fuel combustion, automobiles, spillage of petroleum products, and waste incinerators are significant sources of PAHs into the environment. Tobacco cigarette smoking is a significant source of PAH exposure to smokers and secondary smokers. PAHs generated during anthropogenic combustion activities are primarily transported via atmospheric deposition [13, 14]. Petroleum refining and transport activities are major contributors to localized loadings of PAHs into the environment. Such loadings may occur through discharge of industrial effluents and through accidental release of raw and refined products [9].

Heterocyclic compounds including dibenzothiophene and carbazole are components of creosote, crude oils, and shale oils and often co-exist in the environment with PAHs and other aromatic compounds [15]. Dibenzothiophene is a sulfur heterocyclic compound and is quite persistent in the environment. Little information about dibenzothiophene toxicity is available in the literature. Carbazole, a nitrogen heterocycle, is carcinogenic and toxic [16]. Dibenzofuran and its substituted analogues are found in several woody plants as stress chemicals, so called phytoalexins [17]. However, most of the environmental concerns with dibenzofuran are related to its halogenated analogues, especially its chloro/bromo derivatives.

Persistent organic pollutants (POPs) are among the most concerned environmental pollutants because they persist in the environment, bioaccumulate through the food web, and pose a risk of causing adverse effects to the environment and human health. POPs are also referred to as persistent, bioaccumulative and toxic chemicals (PBTs). POPs include aldrin, brominated flame retardants, chlordane, DDT, dieldrin, endrin, mirex, organometallic compounds such as tributyltin, PAHs, heptachlor, hexachlorobenzene, polychlorinated biphenyls (PCBs), polychlorinated dibenzo-*p*-dioxins (PCDDs), polychlorinated dibenzofurans (PCDFs), and toxaphene. PCDD/Fs are formed unintentionally from human activities. One of the main sources of PCDD/Fs is municipal waste incinerators [18]. PCDD/Fs formed are absorbed on the fly ash, and then enter into the environment. Therefore, the fly ash has been considered as a harmful waste causing environmental pollution. Yang *et al.* [19] suggested an efficient catalytic detoxification method for PCDD/Fs in fly ash.

Alkyl PAHs (e.g., methylnaphthalene) have increasingly become an environmental concern. Because alkyl substitution causes a substantial decrease of water solubility, alkyl PAHs tend to be bioaccumulative. They are abundant in fossil fuels, crude oil and petroleum derived products. Boylan and Tripp [20] identified a large number of aromatic hydrocarbons (e.g., alkyl-benzenes and naphthalenes) in seawater extracts of several crude oils and kerosene. Alexander *et al.* [21] suggested that methylnaphthalenes may be useful indicators of thermal maturity of sedimentary organic matter. Methylnaphthalenes in a petroleum oil fraction can be used as active ingredient of repellent to control mosquitoes [22]. Alkylbenz[*a*]anthracene, especially 7,12-dimethylbenz[*a*]anthracene, is a potent carcinogen in rodent skin and mammal cells [23].

## 3. Aerobic Bacterial Catabolic Pathways for Selected PAHs

### 3.1. Naphthalene

Naphthalene has often been used as a model compound to investigate the ability of bacteria to degrade PAHs because it is the simplest and the most soluble PAH [24]. Therefore, information of bacterial degradation of naphthalene has been used to understand and predict pathways in the degradation of three- or more ring PAHs. Many bacteria that have been isolated and utilize naphthalene as a sole source of carbon and energy belong to the genera *Alcaligenes*, *Burkholderia*, *Mycobacterium*, *Polaromonas*, *Pseudomonas*, *Ralstonia*, *Rhodococcus*, *Sphingomonas*, and *Streptomyces* (Table 1) [3, 10, 25–36, 152, 153].

**Scheme 1.**
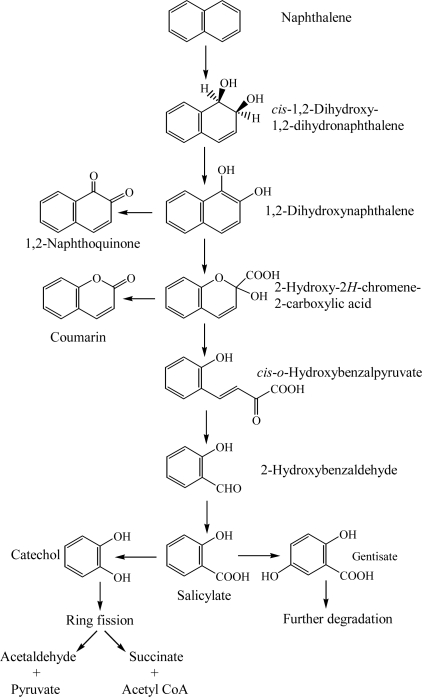
Proposed catabolic pathways of naphthalene by bacteria [24–27, 33].

Degradation of naphthalene starts through the multicomponent enzyme, naphthalene dioxygenase, attack on the aromatic ring to form *cis*-(1R, 2S)-dihydroxy-1,2-dihydronaphthalene (*cis*-naphthalene dihydrodiol) (Scheme 1) [24, 37]. The *cis*-naphthalene dihydrodiol formed by naphthalene dioxygenase is subsequently dehydrogenated to 1,2-dihydroxynaphthalene by a *cis*-dihydrodiol dehydrogenase [24, 25]. Subsequently, 1,2-dihydroxynaphthalene is metabolized to salicylate via 2-hydroxy-2*H*-chromene-2-carboxylic acid, *cis*-*o*-hydroxybenzalpyruvate, and 2-hydroxy-benzaldehyde (Scheme 1) [24, 26, 27, 33]. Also, 1,2-dihydroxynaphthalene is nonenzymatically oxidized to 1,2-naphthaquinone [25]. Salicylate is typically decarboxylated to catechol, which is further metabolized by ring fission in *meta*- and *ortho*-pathways. Fuenmayor *et al.* [29] reported that salicylate is converted to gentisate by salicylate-5-hydroxylase. Recently, Jouanneau *et al.* [31] purified the salicylate 1-hydroxylase from *Sphingomonas* sp. strain CHY-1 and characterized its biochemical and catalytic properties.

The bacterial degradation of naphthalene has been well characterized for the catabolic enzyme system encoded by the plasmid NAH7 in *Pseudomonas putida* G7 [27, 37, 38]. NAH7 has two operons that contain the structural genes for naphthalene degradation. One operon contains the gene for the upper catabolic pathway encoding the enzymes necessary for the conversion of naphthalene to salicylate. The second operon contains the gene for the lower catabolic pathway encoding the enzymes necessary for the metabolism of salicylate through the catechol meta-cleavage pathway to pyruvate and acetaldehyde [24, 27, 37, 38]. The entire sequence structure of plasmid NAH7 (82,232 bp) was determined by Sota *et al*. [154]. Also, the complete 83,042 bp sequence of the circular naphthalene degradation plasmid pDTG1 from *Pseudomonas putida* NCIB 9816–4 was determined by Dennis and Zylstra [155]. Parales *et al.* [39] reported that aspartate 205 in the catalytic domain of naphthalene dioxygenase is a necessary residue in the major pathways of electron transfer to mononuclear iron at the active site.

### 3.2. Fluorene

**Scheme 2.**
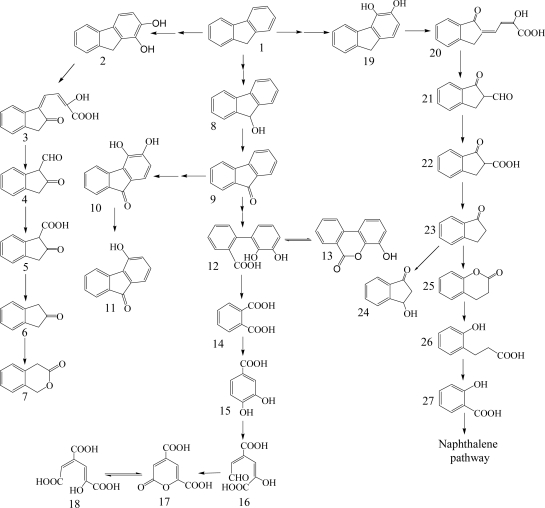
Proposed catabolic pathways of fluorene by bacteria [26, 40, 41, 43, 45, 46]. *Compound designations*: 1, fluorene; 2, fluorene-1,2-diol; 3, 2-hydroxy-4-(2-oxo-indan-1-ylidene)-2-butenoic acid; 4, 1-formyl- 2-indanone; 5, 2-indanone-1-carboxylic acid; 6, 2-indanone; 7, 3-chromanone; 8, 9-fluorenol; 9, 9-fluorenone; 10, 3,4-dihydroxy-9-fluorenone; 11, 4-hydroxy-9-fluorenone; 12, 2’,3’-dihydroxy-biphenyl-2-carboxylic acid; 13, 8-hydroxy-3,4-benzocoumarin; 14, phthalate; 15, protocatechuic acid; 16, 4-carboxy-2-hydroxymuconate- 6-semialdehyde; 17, 2-pyrone-4,6-dicarboxylic acid; 18, 4-carboxy-2-hydroxymuconic acid; 19, fluorene-3,4-diol; 20, 2-hydroxy-4-(1-oxo-indan-2-ylidene)-2-butenoic acid; 21, 2-formyl-1-indanone; 22, 1-indanone-2-carboxylic acid; 23, 1-indanone; 24, 3-hydroxy- 1-indanone; 25, 2-chromanone; 26, 3-(2-hydroxy-phenyl)-propionic acid; 27, salicylic acid.

Fluorene having three rings is a major constituent of fossil fuels and coal derivatives. Several bacteria able to use fluorene as their sole source of carbon and energy have been isolated and are in the genera of *Arthrobacter*, *Brevibacterium*, *Burkholderia*, *Mycobacterium*, *Pseudomonas* and *Sphingomonas* (Table 1) [26, 40–45]. Three major catabolic pathways are shown in Scheme 2. The initial 1,2-dioxygenation of fluorene forms fluorene-1,2-diol that is further transformed to 3-chromanone *via* 2-hydroxy-4-(2-oxo-indan-1-ylidene)-2-butenoic acid, 1-formyl-2-indanone, 2-indanone-1-carboxylic acid, and 2-indanone [41, 46]. The second pathway begins at an initial 3,4-dioxygenation of fluorene leading to salicylate formation through 2-hydroxy-4-(1-oxo-indan-2-ylidene)-2-butenoic acid, 2-formyl-1-indanone, 1-indanone-2-carboxylic acid, 1-indanone, 2-chromanone, and 3-(2-hydroxy-phenyl)-propionic acid [41, 43]. The third pathway starts from C-9 monooxygenation in *Brevibacterium* sp. DPO1361 and *Pseudomonas* sp. F274. This pathway is only productive if a subsequent angular carbon dioxygenation occurs, leading to the formation of phthalate that is further transformed to protocatechuate (Scheme 2) [40, 42, 44, 45, 47]. Several genes involved in the degradation of fluorene to phthalate were characterized in *Terrabacter* sp. DBF63 by Habe *et al.* [48].

### 3.3. Phenanthrene

Phenanthrene, a three aromatic ring system, is found in high concentrations in PAH-contaminated sediments, surface soils, and waste sites [49]. Bacterial degradation of phenanthrene has been extensively studied. A variety of bacterial strains in *Acidovorax*, *Arthrobacter*, *Brevibacterium*, *Burkholderia*, *Comamonas*, *Mycobacterium*, *Pseudomonas*, and *Sphingomonas* have been isolated and have the ability to utilize phenanthrene as a sole carbon and energy source (Table 1) [10, 26, 32, 34, 36, 40, 49–59].

**Scheme 3.**
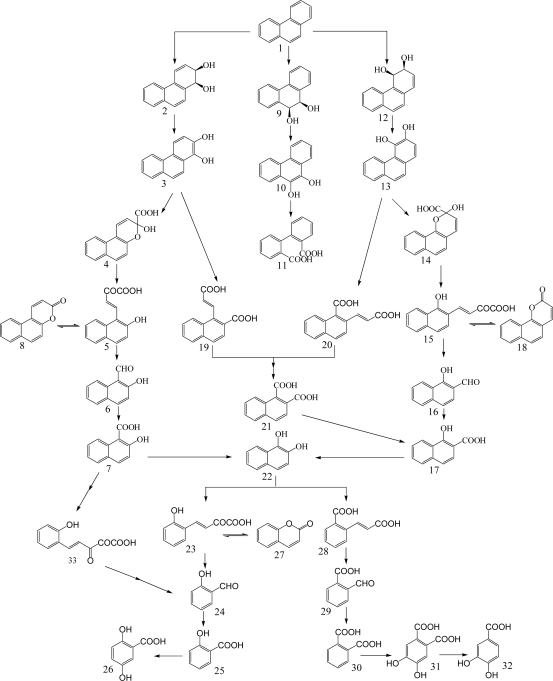
Proposed catabolic pathways of phenanthrene by bacteria [24, 49, 55, 57, 58, 63, 139, 150]. *Compound designations*: 1, phenanthrene; 2, *cis*-1,2-dihydroxy-1,2-dihydrophenanthrene; 3, 1,2- dihydroxyphenanthrene; 4, 3-hydroxy-3*H*-benzo[*f*]chromene-3-carboxylic acid; 5, 4-(2-hydroxy-naphthalen-1-yl)-2-oxo-but-3-enoic acid; 6, 2-hydroxy-naphthalene-1-carbaldehyde; 7, 2-hydroxy-1-naphthoic acid; 8, 5,6-benzocoumarin; 9, *cis*-9,10-dihydroxy-9,10-dihydrophenanthrene; 10, 9,10-dihydroxyphenanthrene; 11, 2,2’-diphenic acid; 12, *cis*-3,4-dihydroxy-3,4-dihydrophenanthrene; 13, 3,4-dihydroxyphenanthrene; 14, 2-hydroxy-2*H*-benzo[*h*]chromene-2-carboxylic acid; 15, 4-(1-hydroxynaphthalen-2-yl)-2-oxo-but-3-enoic acid; 16, 1-hydroxy-naphthalene-2-carbaldehyde; 17, 1-hydroxy-2-naphthoic acid; 18, 7,8-benzocoumarin; 19, 1-(2-carboxy-vinyl)-naphthalene-2-carboxylic acid; 20, 2-(2-carboxy-vinyl)-naphthalene-1-carboxylic acid; 21, naphthalene-1,2-dicarboxylic acid; 22, naphthalene-1,2-diol; 23, 2-hydroxybenzalpyruvic acid; 24, salicylic aldehyde; 25, salicylic acid; 26, gentisic acid; 27, coumarin; 28, 2-carboxycinnamic acid; 29, 2-formylbenzoic acid; 30, phthalic acid; 31, 3,4-dihydroxyphthalic acid; 32, protocatechuic acid; 33, *trans*-2,3-dioxo-5-(2’-hydroxyphenyl)-pent-4-enoic acid.

Phenanthrene contains bay- and K-regions able to form an epoxide, which is suspected to be an ultimate carcinogen [56, 60]. Therefore, it is used as a model substrate for studies on the catabolism of bay- and K-region containing carcinogenic PAHs such as benzo[*a*]pyrene, benzo[*a*]anthracene, and chrysene [56]. In general, bacterial degradation of phenanthrene is initiated by 3,4-dioxygenation to yield *cis*-3,4-dihydroxy-3,4-dihydrophenanthrene, which undergoes enzymatic dehydrogenation to 3,4-dihydroxyphenanthrene (Scheme 3) [57, 58, 61]. The diol is subsequently catabolized to naphthalene-1,2-diol through both *ortho*-cleavage to form 2-(2-carboxy-vinyl)-naphthalene-1-carboxylic acid and *meta*-cleavage to form 4-(1-hydroxy-naphthalen-2-yl)-2-oxo-but-3-enoic acid [57].

Recently, Seo *et al.* [57] elucidated that the *ortho*-cleavage product, 2-(2-carboxy-vinyl)-naphthalene-1-carboxylic acid, is degraded to naphthalene-1,2-diol through naphthalene-1,2-dicarboxylic acid and 1-hydroxy-2-naphthoic acid. Pagnout *et al.* [62] isolated and characterized a gene cluster involved in phenanthrene degradation by 3,4-phenanthrene dioxygenation and *meta*-cleavage. It is possible that 1,2-dioxygenation of phenanthrene forms *cis*-1,2-dihydroxy-1,2-dihydrophenanthrene, which undergoes enzymatic dehydrogenation to 1,2-dihydroxyphenanthrene (Scheme 3). This diol is also subsequently catabolized to naphthalene-1,2-diol through both *ortho*- and *meta*-cleavages. In general, phenanthrene-1,2- and 3,4-diols mainly undergo *meta*-cleavage due to the rapid accumulation of 5,6- and 7,8-benzocoumarin [57]. Naphthalene-1,2-diol converged from 1-hydroxy-2-naphthoic acid and 2-hydroxy-1-naphthoic acid is further degraded in a phthalic acid pathway through *ortho*-cleavage and a salicylic acid pathway through *meta*-cleavage [55, 57, 58]. Mallick *et al.* [63] reported that a novel *meta*-cleavage of 2-hydroxy-1-naphthoic acid to form *trans*-2,3-dioxo-5-(2’-hydroxyphenyl)-pent-4-enoic acid in *Staphylococcus* sp. PN/Y. Interestingly, phenanthrene degradation starts from 9,10-dioxygenase to yield phenanthrene *cis*-9,10-dihydrodiol that is further catabolized to 2,2’-diphenic acid *via* 9,10-dihydroxyphenanthrene [49, 57, 61].

### 3.4. Fluoranthene

Fluoranthene, a four-ring PAH, is one of the principal PAHs in the environment. Bacterial transformation of fluoranthene has been reported (Table 1) [40, 51, 64–73]. *Mycobacterium* has been extensively studied and is a well-known genus to mineralize high molecular weight PAHs such as fluoranthene, pyrene, and benzo[*a*]pyrene [4, 70, 74–79]. Also, strains in the genera *Burkholderia*, *Pasteurella*, *Rhodococcus*, *Sphingomonas*, and *Stenotrophomonas* have been isolated to degrade fluoranthene, using it as a sole carbon and energy sources [64, 66, 71, 72, 80, 81].

**Scheme 4.**
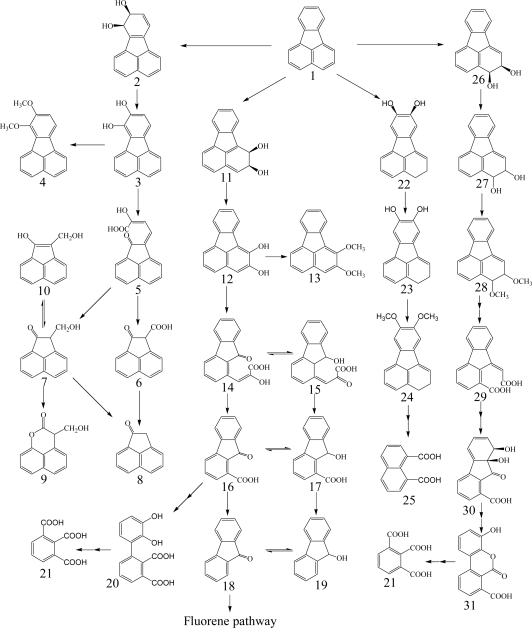
Proposed catabolic pathways of fluoranthene by bacteria [3, 67–70, 85, 147]. *Compound designations*: 1, fluoranthene; 2, *cis*-7,8-fluoranthene dihydrodiol; 3, 7,8-dihydroxy-fluoranthene; 4, 7,8-dimethoxyfluoranthene; 5, 2-hydroxy-4-(2-oxo-2*H*-acenaphthylen-1-ylidene)-but-2-enoic acid; 6, 2-oxo-acenaphthene-1-carboxylic acid; 7, 2-hydroxylmethyl-2*H*-acenaphthylen-1-one; 8, 1-acenaphthenone; 9, 3-hydroxymethyl-3*H*-benzo[*de*] chromen-2-one; 10, 2-hydroxymethyl-acenaphthylen-1-ol; 11, *cis*-1,2-fluoranthene dihydrodiol; 12, 1,2-dihydroxy-fluoranthene; 13, 1,2-dimethoxyfluoranthene; 14, 9-fluorenone-1-(carboxy-2-hydroxy-1-propenol); 15, 9-fluorenol-1-carboxy-3-propenyl-2-one; 16, 9-fluorenone-1-carboxylic acid; 17, 9-fluorenol-1-carboxylic acid; 18, 9-fluorenone; 19, 9-fluorenol; 20, 2’,3’-dihydroxybiphenyl-2,3-dicarboxylic acid; 21, benzene-1,2,3-tricarboxylic acid; 22, *cis*-8,9-fluoranthene dihydrodiol; 23, 8,9-dihydroxy-fluoranthene; 24, 8,9-dimethoxyfluoranthene; 25, naphthalene-1,8-dicarboxylic acid; 26, *cis*-2,3-fluoranthene dihydrodiol; 27, 2,3-dihydroxy-fluoranthene; 28, 2,3-dimethoxyfluoranthene; 29, 9-carboxymethylene-9*H*-fluorene-1-carboxylic acid; 30, *cis*-1,9a-dihydroxy-1-hydrofluorene-9-one-8-carboxylic acid; 31, 4-hydroxybenzochromene-6-one-7-carboxylic acid.

Bacterial degradation of fluoranthene is generally initiated by 1,2- or 7,8-dioxygenation to form *cis*-1,2- or *cis*-7,8-fluoranthene dihydrodiol, respectively (Scheme 4). These two dihydrodiols are dehydrogenated to 1,2- or 7,8-dihydroxyfluoranthene, respectively. 7,8-Dihydroxy-fluoranthene is further transformed *via meta*-cleavage to 1-acenaphthenone and 3-hydroxymethyl-3*H*-benzo[*de*]-chromen-2-one through 2-hydroxyl-4-(2-oxo-2*H*-acenaphthylen-1-ylidene)-but-2-enoic acid, 2-hydroxylmethyl-2*H*-acenaphthylen-1-one, and 2-oxo-acenaphthene-1-carboxylic acid. Kelley *et al.* [67] reported that the dehydrogenation of a transient *cis*-7,8-fluoranthene dihydrodiol to form 7,8-dihydroxyfluoranthene with *O*-methylation at the 7-position would form 7-methoxy-8-hydroxy-fluoranthene. However, Lee *et al.* [68] suggested that there are four possible initial dioxygenation at 1,2-, 2,3-, 7,8-, and 8,9-positions of fluoranthene and four possible dimethoxyfluoranthenes in *Mycobacterium* sp. JS14. 1,2-Dihydroxy-fluoranthene is also further degraded by *meta*-cleavage to 9-fluorenone through 9-fluorenone-1-(carboxy-2-hydroxy-1-propenol) and 9-fluorenone-1-carboxylic acid which can be converted by protonation to 9-fluorenol-1-carboxy-3-propenyl-2-one and 9-fluorenol-1-carboxylic acid, respectively. The conversion of 9-fluorenone to 9-fluorenol is also possible.

Rehmann *et al.* [69] reported 2,3-dioxygenation of fluoranthene and found five metabolites, namely *cis*-2,3-fluoranthene dihydrodiol, 9-carboxymethylene-9*H*-fluorene-1-carboxylic acid, *cis*-1,9a-dihydroxy-1-hydrofluorene-9-one-8-carboxylic acid, 4-hydroxybenzochromene-6-one-7-carboxylic acid, and benzene-1,2,3-tricarboxylic acid. *cis*-2,3-Fluoranthene dihydrodiol is catabolized by *ortho*-cleavage to benzene-1,2,3-tricarboxylic acid *via* 9-carboxymethylene-9*H*-fluorene-1-carboxylic acid, *cis*-1,9a-dihydroxy-1-hydrofluorene-9-one-8-carboxylic acid, and 4-hydroxybenzo-chromene-6-one-7-carboxylic acid. Recently, Lee *et al.* [68] proposed 8,9-dioxygenation of fluoranthene from the detection of 8,9-dimethoxyfluoranthene. Also, they detected 25 proteins related to fluoranthene catabolism in *Mycobacterium* sp. JS14 using 1-D SDS PAGE, 2-D SDS PAGE, and nano-LC/MS/MS.

### 3.5. Pyrene

Pyrene possessing four benzene rings is a byproduct of gasification processes and other incomplete combustion processes. Many bacterial isolates capable of degrading pyrene have been studied (Table 1). *Mycobacterium* as Gram-positive species has been most widely studied for degrading pyrene by using it as a sole carbon and energy source [4, 34, 35, 51, 61, 75, 78, 79, 83–85]. *Mycobacterium* spp. are known to have high cell surface hydrophobicity and adhere to the emulsified solvent droplets [83]. Other pyrene degrading strains isolated include *Rhodococcus* sp. [86], *Bacillus cereus* [87], *Burkholderia cepacia* [80], *Cycloclasticus* sp. P1 [88], *Pseudomonas fluorescens* [64], *Pseudomonas stutzeri* [87], *Sphingomonas* sp. VKM B-2434 [26], *Sphingomonas paucimobilis* [89], and *Stenotrophomonas maltophilia* [64, 66, 90].

**Scheme 5.**
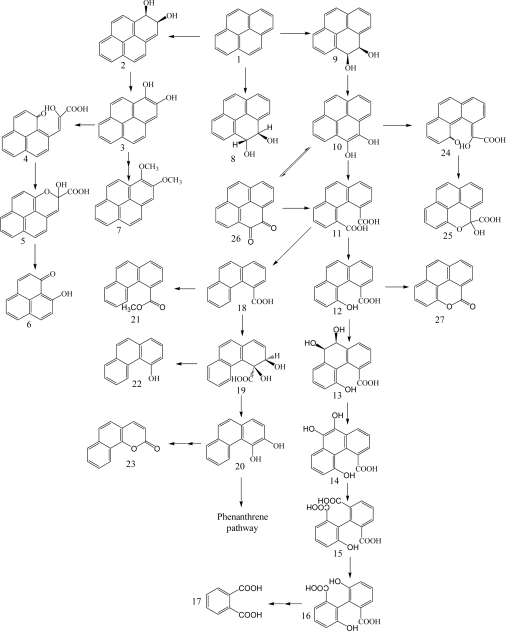
Proposed catabolic pathways of pyrene by bacteria [3, 9, 61, 76, 78, 82, 85, 86, 91, 139]. *Compound designations*: 1, pyrene; 2, pyrene-*cis*-1,2-dihydrodiol; 3, pyrene-1,2-diol; 4, 2-hydroxy-3-(perinaphthenone-9-yl)-propenic acid; 5, 2-hydroxy-2*H*-1-oxa-pyrene-2-carboxylic acid; 6, 4-hydroxyperinaphthenone; 7, 1,2-dimethoxypyrene; 8, pyrene-*trans*-4,5-dihydrodiol; 9, pyrene-*cis*-4,5-dihydrodiol; 10, pyrene-4,5-diol; 11, phenanthrene-4,5-dicarboxylic acid; 12, 4-carboxyphenanthrene-5-ol; 13, 4-carboxy-5-hydroxy-phenanthrene-9,10-dihydrodiol; 14, 4-carboxyphenanthrene-5,9,10-triol; 15, 2,6,6’-tricarboxy-2’-hydroxybiphenyl; 16, 2,2’-dicarboxy-6,6’-dihydroxybiphenyl; 17, phthalic acid; 18, 4-phenantroic acid; 19, 3,4-dihydroxy-3,4-dihydro-phenanthrene-4-carboxylic acid; 20, phenanthrene-3,4-diol; 21, 4-phenanthroic acid methyl ester; 22, 4-hydroxyphenanthrene; 23, 7,8-benzocoumarin; 24, 2-hydroxy-2-(phenanthrene-5-one-4-enyl)-acetic acid; 25, 5-hydroxy-5*H*-4-oxa-pyrene-5-carboxylic acid; 26, pyrene-4,5-dione; 27, 4-oxa-pyrene-5-one.

Heitkamp *et al.* [76] found the three products of ring oxidation, pyrene-*cis*-4,5-dihydrodiol, pyrene-*trans*-4,5-dihydrodiol, and pyrenol, and four products of ring fission (Scheme 5), 4-hydroxyperinaphthenone, 4-phenanthroic acid, phthalic acid, and cinnamic acid by multiple analyses, including UV, infrared, mass spectrometry, NMR, and GC. The formation of pyrene-*cis*-4,5-dihydrodiol by dioxygenase and pyrene-*trans*-4,5-dihydrodiol by monooxygenase suggested multiple initial oxidative attacks on pyrene. Pyrene-1,2-diol derived from the dioxygenation at pyrene 1,2-C positions is metabolized to 4-hydroxyperinaphthenone *via cis*-2-hydroxy-3-(perinaphthenone-9-yl)-propenic acid and 2-hydroxy-2*H*-1-oxa-pyrene-2-carboxylic acid (Scheme 5). Kim and Freeman [61] found 1,2-dimethoxypyrene as a subproduct of pyrene-1,2-diol. Pyrene-4,5-diol is degraded by *ortho*-cleavage to phenanthrene-4,5-dicarboxylic acid, which is further metabolized through phenanthrene-3,4-diol pathway and 6,6’-dihydroxy-2,2’-biphenyl-dicarboxylic acid pathway. *Meta*-cleavage of pyrene-4,5-diol lead to 5-hydroxy-5*H*-4-oxa-pyrene-5-carboxylic acid *via* 2-hydroxy-2-(phenanthrene-5-one-4-enyl)-acetic acid (Scheme 5). A novel metabolite, 6,6’-dihydroxy-2,2’-biphenyl-dicarboxylic acid, was identified by Vila *et al.* [85] from the degradation of pyrene by *Mycobacterium* sp. strain AP1. Liang *et al.* [91] reported pyrene-4,5-dione formation and identified almost all the enzymes required during the initial steps of pyrene degradation in *Mycobacterium* sp. KMS. Kim *et al.* [92] identified 27 enzymes necessary for constructing a complete pathway for pyrene degradation from both genomic and proteomic data.

### 3.6. Benzo[a]pyrene

Benzo[*a*]pyrene, a five aromatic ring PAH, is one of the most potent carcinogenic PAHs. Benzo[*a*]pyrene has low water solubility (0.0023 mg/L) and a high octanol/water partition coefficient (LogK_ow_, 6.06), which is related to high recalcitrance to microbial degradation. It also has relatively low abundance in environmental samples which limits its catabolism by bacterial assemblages. The concentration of benzo[*a*]pyrene as well as other PAHs in contaminated soils in industrial sites can vary depending on the industrial activities associated with the site. To date, there is only limited information regarding the bacterial degradation of PAHs with five or more rings. There are several bacterial species capable of benzo[*a*]pyrene degradation including *Mycobacterium* sp. [74, 79, 93], *Sphingomonas paucimobilis* [94], and *Stenotrophomonas maltophilia* [95] (Table 1). Juhasz *et al.* [66] found that *S. maltophilia* VUN 10,003 can degrade benzo[*a*]pyrene by 22% for 14 days incubation. A *S. paucimobilis* EPA 505 grown on fluoranthene degraded 33% of benzo[*a*]pyrene [94].

**Scheme 6.**
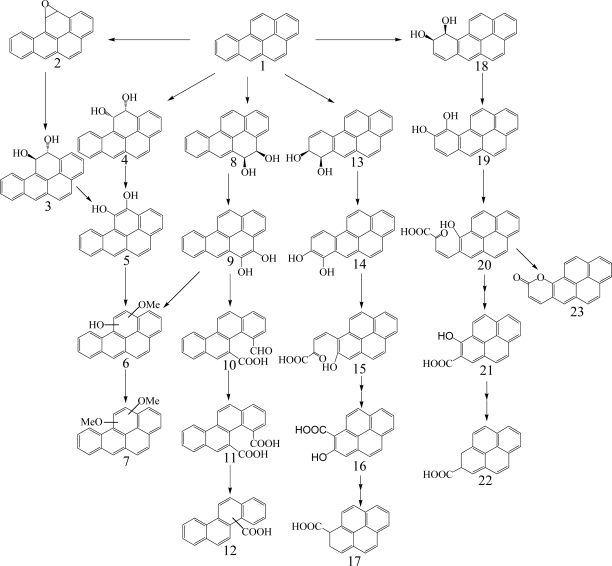
Proposed catabolic pathways of benzo[*a*]pyrene by bacteria [79, 99, 100]. *Compound designations*: 1, benzo[*a*]pyrene; 2, benzo[*a*]pyrene-11,12-epoxide; 3, benzo[*a*]pyrene trans-11,12-dihydrodiol; 4, benzo[*a*]pyrene cis-11,12-dihydrodiol; 5, 11,12-dihydroxybenzo[*a*]pyrene; 6, hydroxymethoxybenzo[*a*]pyrene; 7, dimethoxybenzo[*a*]pyrene; 8, benzo[*a*]pyrene cis-4,5-dihydrodiol; 9, 4,5-dihydroxybenzo[*a*]pyrene; 10, 4-formylchrysene-5-carboxylic acid; 11, 4,5-chrysene-dicarboxylic acid; 12, chrysene-4 or 5-carboxylic acid; 13, benzo[*a*]pyrene *cis*-7,8-dihydrodiol; 14, 7,8-dihydroxybenzo[*a*]pyrene; 15, *cis*-4-(7-hydroxypyrene-8-yl)-2-oxobut-3-enoic acid; 16, pyrene-7-hydroxy-8-carboxylic acid; 17, 7,8-dihydro-pyrene-8-carboxylic acid; 18, benzo[*a*]pyrene *cis*-9,10-dihydrodiol; 19, 9,10-dihydroxybenzo[*a*]pyrene; 20, *cis*-4-(8-hydroxypyrene-7-yl)-2-oxobut-3-enoic acid; 21, pyrene-8-hydroxy-7-carboxylic acid; 22, 7,8-dihydro-pyrene-7-carboxylic acid; 23, 10-oxabenzo[*def*]chrysene-9-one.

Gibson *et al.* [96] first identified *cis*-7,8 and 9,10-benzo[*a*]pyrene-dihydrodiol formed in *Beijerinckia* sp. strain B1, except ring cleavage metabolites (Scheme 6). Schneider *et al.* [79] reported that *Mycobacterium* sp. strain RJGII-135 is capable of transforming benzo[*a*]pyrene to initial ring oxidation and ring cleavage metabolites. They found one ring oxidation metabolite, benzo[*a*]pyrene *cis*-7,8-dihydrodiol, and three ring cleavage metabolites, 4,5-chrysene-dicarboxylic acid, *cis*-4-(8-hydroxypyrene-7-yl)-2-oxobut-3-enoic acid or *cis*-4-(7-hydroxypyrene-8-yl)-2-oxobut-3-enoic acid, and 7,8-dihydro-pyrene-7-carboxylic acid or 7,8-dihydro-pyrene-8-carboxylic acid (Scheme 6). These metabolites indicate that the initial enzymatic attack occurs at the 4,5-, 7,8-, and 9,10-positions of benzo[*a*]pyrene.

The genes encoding the α-subunit of the PAH-ring hydroxylating dioxygenases involved in the initial step of metabolism of PAH in bacteria were recently quantified and characterized by Cebron *et al.* [97] and Lozada *et al.* [98]. Benzo[*a*]pyrene *cis*-7,8-dihydrodiol is further catabolized *via meta*-cleavage to 7,8-dihydro-pyrene-8-carboxylic acid through *cis*-4-(7-hydroxypyrene-8-yl)-2-oxobut-3-enoic acid. Benzo[*a*]pyrene *cis*-9,10-dihydrodiol is degraded *via meta*-cleavage to 7,8-dihydro-pyrene-7-carboxylic acid through *cis*-4-(8-hydroxypyrene-7-yl)-2-oxobut-3-enoic acid. In addition, 10-oxabenzo[*def*]-chrysene-9-one formed by dehydration of benzo[*a*]pyrene *cis*-9,10-dihydrodiol to give *cis*-4-(8-hydroxypyrene-7-yl)-2-oxobut-3-enoic acid with subsequent *meta*-cleavage and aromatic ring closure [99]. 4,5-Chrysene-dicarboxylic acid is transformed from 4-formylchrysene-5-carboxylic acid formed by the *ortho*-cleavage of 4,5-dihydroxy benzo[*a*]pyrene. Moody *et al.* [99] reported new degradation pathways of benzo[*a*]pyrene in *Mycobacterium vanbaalehii* PYR-1. The new pathways are initiated by dioxygenase and monooxygenase at the 11,12-positions of benzo[*a*]pyrene molecule to form benzo[*a*]pyrene *cis*-11,12-dihydrodiol and benzo[*a*]pyrene *trans*-11,12-dihydrodiol, respectively. Benzo[*a*]pyrene *cis*-11,12-dihydrodiol is further catabolized to dimethoxybenzo[*a*]pyrene *via* hydroxymethoxybenzo[*a*]pyrene. The formation of benzo[*a*]pyrene *trans*-11,12-dihydrodiol is due to cytochrome P-450 acting to form benzo[*a*]pyrene 11,12-epoxide with subsequent hydrolysis by epoxide hydrolase. Recently, Rentz *et al.* [100] identified two novel ring cleavage metabolites, pyrene-8-hydroxy-7-carboxylic acid and pyrene-7-hydroxy-8-carboxylic acid, formed by *Sphingomonas yanoikuyae* JAR02.

## 4. Aerobic Bacterial Catabolism of Aromatic Heterocycles

Various types of heterocycles containing oxygen, nitrogen, and sulfur are found in the environment, originating from anthropogenic or natural sources. Dibenzofurans, dibenzodioxins, and dibenzothiophene are among the most important environmental pollutants and are well reviewed [2, 101, 102]. Therefore, only aerobic bacterial degradation of non-halogenated heterocyclic aromatics is briefly discussed in this review to make a general relation with PAH degradation.

**Scheme 7.**
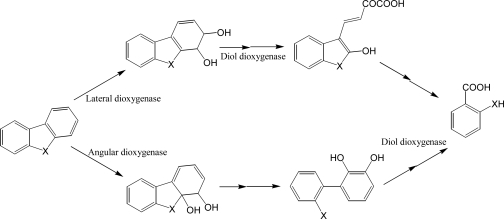
Simplified bacterial catabolic pathways of dibenzofuran (X= O) and carbazole (X = S).

Many bacterial species have been reported to decompose dibenzofurans and carbazole, a structural analogue with nitrogen instead of oxygen [103–108], although some white rot fungi are well known to decompose halogenated dibenzofurans. Guo *et al.* [106] isolated a stable carbazole-degrading microbial consortium consisting of *Chryseobacterium* sp. NCY and *Achromobacter* sp. NCW. As for the degradation of unsubstituted PAHs, bacterial catabolism of dibenzofurans starts at insertion of two oxygen atoms catalyzed by dioxygenases. Although many bacterial PAH dioxygenases are evolutionarily related to phenylpropionate dioxygenase, striking diversities have been reported in catalytic activity, mechanisms, regulations, and substrates of these important enzymes. In short, initial reactions of dibenzofuran and carbazole can be classified into angular and lateral dioxygenation, which may be catalyzed by different enzymes (Scheme 7). These enzymes were found in Gram-positive and negative bacteria [109–112]. Some bacterial dioxygenases from *Pseudomonas* sp. CA10 and *Sphingomonas wittichii* RW1, for example, catalyze predominantly angular insertion of oxygen [109, 112] while the commonly known naphthalene dioxygenase from *Pseudomonas* sp. NCIB 9816-4 catalyzes only lateral dioxygenation [113]. According to the metabolite analyses accompanied by biochemical studies, angular and lateral dioxygenases were considered from different origins.

However, recent studies with cloned dioxygenase from *Norcardioides aromaticivorans* IC177 revealed that some dioxygenase can catalyze both reactions [110]. It is well known that PAH dioxygenases can catalyze various reactions, including reduction, mono- and di-oxygenation [113]. In addition to the multiple reactions with specific dioxygenases, current genomic or proteomic research with several PAH degrading bacteria (e.g., *Burkholderia* spp. and *Mycobacterium* spp.) revealed that multiple dioxygenases, probably playing different roles in PAH degradation, exist in a single bacterium [91, 114, 115]. For example, metabolite profiling of culture supernatant of *Janibacter* sp. YY1 treated with dibenzofuran showed that it may have both angular and lateral dioxygenases [116]. Multiple catabolic pathways can produce various metabolites with structural diversities, which probably are metabolized by different types of enzymes. For example, Nojiri *et al.* [107] reported that *Terrabacter* sp. DBF63 can metabolize fluorene and dibenzofuran by the same dioxygenase but the metabolites, namely phthalate and salicyclate from fluorene and dibenzofuran, respectively, are further catabolized by different enzymes, of which the related genes are located in different areas of chromosomes.

**Scheme 8.**
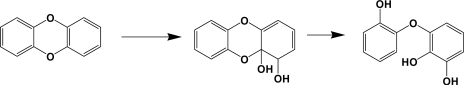
Bacterial catabolic pathways of dibenzo-*p*-dioxin.

Dioxygenases involving in dibenzo-*p*-dioxins degradation catalyze predominantly an angular dioxygenation to produce trihydroxy diphenyl ethers (Scheme 8). PCDDs are well known pollutants with extreme toxicity, especially tetra to hexachloro analogues. Some bacterial species can catabolize up to hexachlorodibenzo-*p*-dioxins [112, 117]. In comparison with dibenzofuran catabolism, chlorophthalates or salicylates and chlorophenols are common metabolites of PCDDs [112]. Chlorophenols are well known to be toxic to various organisms, including their degrading bacteria. Toxic effects of chlorometabolites are also reported from the catabolism of PCBs [118]. Although it is not clear how the bacteria can cope with the toxicity of chlorophenols during dioxin metabolism, glutathione or other sulfur-containing primary metabolites may be involved in its detoxification [118].

**Scheme 9.**
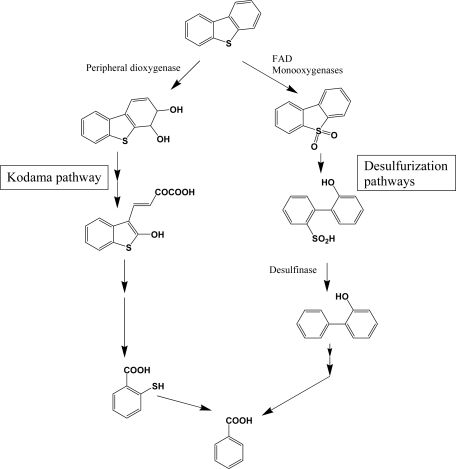
Bacterial catabolic pathways of dibenzothiophene.

Dibenzothiophene and its higher molecular weight analogues are commonly found in higher molecular weight fractions of petroleum, but are not pyrogenic and biogenic PAH components. Although the chemical profiles of sulfur heterocycles are well documented in petroleum products, studies of their biodegradation in petroleum contaminated natural environments are very limited. Because of the economic importance of desulfurization of unprocessed petroleum, detailed research was performed with various bacterial species [119–121]. Catabolism of dibenzothiophene is catalyzed by distinct enzymes in two pathways (Scheme 9). The catabolic branch of initial sulfur oxidation is also called 4S pathway, through which rapid desulfurization can be obtained. Flavin-containing monooxygenases, noted as DszA and DszB (or SoxA and SoxB), are widely distributed in bacteria and catalyze a consecutive addition of single oxygen atom. Consecutive desulfurization is achieved by desulfinase. These monooxygenases require FAD as a cofactor, accompanied by a specific flavin reductase [122]. FAD containing monooxygenases are very common in all biota and catalyze various detoxification steps. For example, Sutherland *et al.* [123] reported a FAD containing monooxygenase of high sequence homology with dibenzothiophene desulfurization enzymes. In general, bacterial species with high-desulfurization activities have all of the enzymes and broad substrate range [120, 121]. However, some bacterial species are defective in some components, which results in accumulation of intermediates. In comparison with desulfurization pathway, the enzymes for lateral dioxygenation and consecutive reactions are very different (Kodama pathway, Scheme 9). Many bacterial species have been reported to metabolize dibenzothiphene through Kodama pathway [105, 108, 113]. Although no detailed research has been done, the structural similarities of the metabolites suggest that common PAH dioxygenase and enzymes in successive steps may be involved in Kodama pathway. According to Resnick and Gibson [113], naphthalene dioxygenase from *Pseudomonas* sp. NCIB 9816-4 can produce dibenzothiophene dihydrodiol, a lateral ring dioxygenation product, which supports the broad action of PAH dioxygenase on sulfur heterocycles.

Among the nitrogen heterocycles, catabolism of carbazole is well documented. However, other heterocycles, including benzoquinolines and phenanthridine are not well studied. In comparison with the other heterocycles, nitrogen heterocycles are well known metal chelators and can easily deactivate various metalloenzymes. Seo *et al.* [108] reported carbazole catabolism in *Arthrobacter* sp. P1-1. Catabolism of benzoquinoline in *Mycobacterium gilvum* LB307T suggests that at least some bacteria can overcome the toxic effect of nitrogen heterocycles [124].

## 5. Bacterial Catabolism of Alky PAHs

Alkyl- and nitro-PAHs are among common substituted PAHs and have substantial toxicities. Substitution on aromatic rings produces several problems in their degradation. The ability of PAH dioxygenase to remove the substitutions is currently the subject of debate. An alkyl branch is not a good leaving group and probably requires additional steps to be removed. Their presence may inhibit proper orientation and accessibility of the PAHs into dioxygenases.

**Scheme 10.**
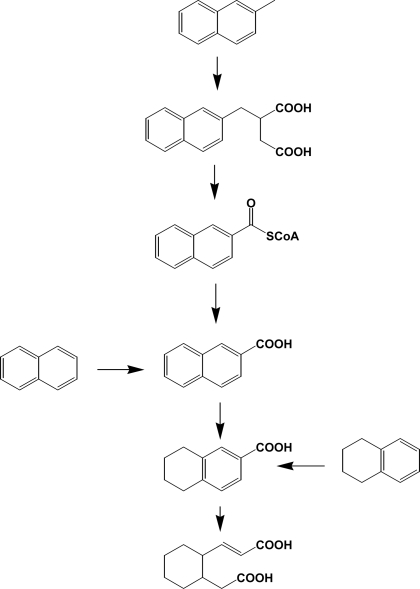
Catabolism of naphthalene, methylnaphthalene, and tetralin by anaerobic bacteria [129–131].

In general, methyl-/ethyl-naphthalenes and phenanthrenes are prevalent contaminants in the environment and, however, limited numbers of studies have been done in relation to bacterial degradation [28, 125, 126]. Catabolism of alkyl PAHs in aerobic bacteria suggests a very diversity of enzymes involved [125]. These include oxidation of methyl group to alcohol, aldehyde, or carboxylic acid, decarboxylation, demethylation, and dioxygenation. However, production of alkyl salicylate or alkylphthalate suggests that the reaction may prefer non-substituted PAH systems. Until recently, research with anaerobic bacteria has been limited to halogenated pollutants such as PCBs, PBDEs, PCDDs/Fs and halogenated solvents [127, 128]. However, PAHs and their alkyl derivatives can be transformed by various anaerobes through novel catabolic pathways (Scheme 10) [129–131, 156]. In comparison with aerobic bacteria initiating dioxygenation, anaerobic bacteria usually insert carboxyl groups from carbon dioxide or succinic acids. Part of the ring is reduced to tetralin type metabolites [130]. The presence of ring cleavage products (e.g., derivatives of cyclohexane) suggests that oxygen molecule or oxygen equivalents may be required in these consecutive catabolic steps (Scheme 10). Metabolism of alkylbenzenes, alkanes, and other hydrocarbons by anaerobic bacteria was well reviewed by Spormann and Widdel [157].

Relatively speaking, studies with nitro-PAHs are much more limited [2, 132]. It is noteworthy that a large amount of nitro-PAHs (e.g., 1-nitropyrene) are produced from automobile exhaustion. It is well known that nitropyrene can be transformed into mutagenic metabolites, including amino-, nitroso-, and hydroxyamin-pyrene. Rafii *et al.* [132] reported that 1-nitropyrene can be transformed into mutagenic 1-aminopyrene by *Mycobacterium* sp. High genotoxic effects of various nitro-PAHs call for more detailed research on their biodegradation.

## 6. Protoemics and Metabolomics in Understanding of Bacterial Degradation of PAHs

Proteomics and metabolomics have been recently employed in studies of environmental microbiology and have shown their high impact on the field of biodegradation and bioremediation [158, 159]. Proteomics is an effective technique to identify proteins and their functions involved in the biodegradation of aromatics while metabolomics can be used to profile degradation products of PAHs and primary metabolites in response to PAH exposures. Intent of a brief discussion of proteomics and metabolomics here is to bring attention to these emerging fields rather than offering a comprehensive review and a long list of references.

A good number of genomic sequences or expressed sequence tags (ESTs) of bacteria are currently available. These include several PAH degrading bacteria in the genus of *Mycobacterium*, *Acinetobacter*, *Arthrobacter*, and *Burkholderia*. Detailed transcriptomes in *Burkholderia xenovorans* LB400 during PCB catabolism were analyzed by microarray techniques [114, 133–135]. The results revealed that large changes at genomic and proteomic levels occurred during the PAH catabolism, compared with the catabolism of natural carbon sources. These include up-regulation of the genes related to a) PAH catabolism, b) removal of reactive oxygen species, c) primary carbon metabolism, including one-carbon metabolism, d) energy production, e.g., ATP synthesis, e) various transporters, and f) synthesis of energy reservoir, e.g., polyphosphate. Many of these phenomena have been demonstrated by specific studies. For example, polyphosphate accumulation during PCB catabolism was confirmed by electron microscopy [136]. The accumulation of polyphosphate and polyphosphate kinase (*Ppk*) under stressed conditions are common in all biota. Up-regulation of superoxide dismutase or catalase/peroxidase is also common phenomena during PAHs or toxic chemical catabolism. The activation of one-carbon metabolism is another common phenomenon in bacteria during xenobiotic metabolism [137]. The exact mechanism of the gene activation is not clear. The up-regulation of one-carbon metabolism may be induced by several factors, including limitation of primary metabolite pools, introduction of one carbon unit, e.g., formate in PAH metabolism, and the loss of concerted regulation of general metabolism. Another common phenomenon is the changes of fatty acid content or composition in bacteria [138]. However, transcriptome analysis usually did not show a significant change in fatty acid metabolism-related genes. Discrepancies between genomic and proteomic data also occur. In general, proteomic analyses in relation to aromatics catabolism are very limited. However, a few proteomic studies of PAH catabolism in *Mycobacterium* spp. have been reported [61, 68, 91, 115, 139]. For example, Lee *et al.* [68] studied fluoranthene catabolism and associated proteins in *Mycobacterium* sp. JS14. An increased expression of 25 proteins related to fluoranthene catabolism is found with 1D-PAGE and nano liquid chromatography tandem MS/MS. Detection of fluoranthene catabolism associated proteins coincides well with its multiple degradation pathways that are mapped via metabolites identified. Kim *et al.* [115] reported the protein profiles of *Mycobacterium vanbaalenii* PYR-1 grown in the presence of pyrene, pyrene-4,5-quinoline, phenanthrene, anthracene, and fluoranthene after two dimensional electrophoresis (2DE) separation of proteins and mass spectrometry analysis. They found PAH-induced proteins (catalase-peroxidase, putative monooxygenase, dioxygenase small subunit, small subunit of naphthalene-inducible dioxygenase, and aldehyde dehydrogenase), carbohydrate metabolism related proteins (enolase, 6-phosphogluconate dehydrogenase, indole-3-glycerol phosphate synthase, and fumarase), DNA translation proteins, heat shock proteins, and energy production protein (ATP synthase). Proteomics studies showed that several *Mycobacterium* species have multiple dioxygenases and related enzymes, which may be involved in different substrates [61, 68, 91]. Those studies also revealed that many of stress related proteins, including enzymes related to reactive oxygen removal and transcriptional regulators are commonly up-regulated during PAH metabolism.

The workflow of proteomics has been extended from qualitative analyses after 1DE or 2DE to quantitative studies. Systematic quantitation of proteomes has become common by using methods such as isotope-coded affinity tag (ICAT), isobaric tags for relative and absolute quantitation (iTRAQ), and stable isotope labeling by essential amino acid culture (SILAC). The labeled proteins or peptides by those methods are analyzed by tandem MS [140, 141]. Recently, Kim *et al.* [142] conducted the proteome analysis of *Pseudomonas putida* KT2440 induced with monocyclic aromatic compounds using 2-DE/MS and cleavable isotope-coded affinity tag (ICAT) to determine the changes of proteins involved in aromatic degradation pathways. After translation, attachment of functional groups such as carbohydrates, lipids, phosphates or sulphur to a protein is referred to as post-translational modification (PTM). PTM extends protein functions and makes proteomes further complicated. It is noteworthy that in tandem mass spectrometry, a combination of collision induced dissociation (CID) and electron transfer dissociation (ETD) is becoming very useful in PTM elucidation. ETD cleaves randomly the peptide backbone while modifications are left intact. CID produces fragment ions for structural determination as well as sensitive and specific detection of a particular molecule.

Among the various system-wide evaluation of bacterial PAH degradation, almost no research has been reported in relation to metabolomics. Keum *et al.* [159] studied comparative metabolic responses of *Sinorhizobium* sp. C4 during degradation of phenanthrene. Comprehensive profiling of metabolites profiles, including polar metabolites, fatty acids and polyhydroxyalkanoates was performed through untargeted metabolome analyses. Large metabolome differences of strain C4 were observed between the cultures with phenanthrene and natural carbon sources. The changes include TCA cycle, pyruvate metabolism, cofactor biosynthesis, fatty acid compositions, and polyhydroxyalkanoate biosynthesis, which indicate metabolic adaptation in response to using phenanthrene as a substrate. Although the analyses of transcriptomes and proteomes can provide significant information indicating metabolic networks, a large number of metabolites interact with each other and thus metabolomes can show much more complex networks than transcriptomes and proteomes. As proteomics is advancing rapidly in environmental microbiology, metabolomics will also become a powerful tool. In conjunction with functional genomics, proteomics and metabolomics are becoming indispensable in elucidation of mechanisms of biodegradation and biotransformation of organic pollutants in the environment, particularly for the situations of multiple chemicals and microbial consortia.

## Figures and Tables

**Table 1:** Isolated bacteria capable of degrading aromatic compounds (incomplete list).

Bacterial species	Strains	Aromatics	References
*Achromobacter* sp.	NCW	CBZ	[106]
*Alcaligenes denitrificans*		FLA	[73]
*Arthrobacter* sp.	F101	FLE	[41]
*Arthrobacter* sp.	P1-1	DBT, CBZ, PHE	[57, 108]
*Arthrobacter sulphureus*	RKJ4	PHE	[56]
*Acidovorax delafieldii*	P4-1	PHE	[56]
*Bacillus cereus*	P21	PYR	[87]
*Brevibacterium* sp.	HL4	PHE	[56]
*Burkholderia* sp.	S3702, RP007, 2A-12TNFYE-5, BS3770	PHE	[32, 50, 143]
*Burkholderia* sp.	C3	PHE	[57]
*Burkholderia cepacia*	BU-3	NAP, PHE, PYR	[10]
*Burkholderia cocovenenans*		PHE	[59]
*Burkholderia xenovorans*	LB400	BZ, BP	[133]
*Chryseobacterium* sp.	NCY	CBZ	[106]
*Cycloclasticus sp.*	P1	PYR	[88]
*Janibacter* sp.	YY-1	DBF, FLE, DBT, PHE, ANT, DD	[116]
*Marinobacter*	NCE312	NAP	[30]
*Mycobacterium* sp.		PYR, BaP	[4, 68, 75, 76, 83, 93, 144]
*Mycobacterium* sp.	JS14	FLA	[68]
*Mycobacterium* sp.	6PY1, KR2, AP1	PYR	[78, 85, 139]
*Mycobacterium* sp.	RJGII-135	PYR,BaA, BaP	[79]
*Mycobacterium* sp.	PYR-1, LB501T	FLA, PYR, PHE, ANT	[49, 67, 70, 77, 87, 145]
*Mycobacterium* sp.	CH1, BG1, BB1, KR20	PHE, FLE, FLA, PYR	[40, 51, 52, 69]
*Mycobacterium flavescens*		PYR, FLA	[65, 82]
*Mycobacterium vanbaalenii*	PYR-1	PHE, PYR, dMBaA	[61, 126]
*Mycobacterium* sp.	KMS	PYR	[84]
*Nocardioides aromaticivorans*	IC177	CBZ	[110]
*Pasteurella* sp.	IFA	FLA	[146]
*Polaromonas naphthalenivorans*	CJ2	NAP	[153]
*Pseudomonas* sp.	C18, PP2, DLC-P11	NAP, PHE	[27, 55, 56]
*Pseudomonas* sp.	BT1d	HFBT	[119]
*Pseudomonas* sp.	B4	BP, CBP	[136]
*Pseudomonas* sp.	HH69	DBF	[104]
*Pseudomonas* sp.	CA10	CBZ, CDD	[109]
*Pseudomonas* sp.	NCIB 9816-4	FLE, DBF, DBT	[113]
*Pseudomonas* sp.	F274	FLE	[47]
*Pseudomonas paucimobilis*		PHE	[73]
*Pseudomonas vesicularis*	OUS82	FLE	[73]
*Pseudomonas putida*	P16, BS3701, BS3750, BS590-P, BS202-P1	NAP, PHE	[33, 50]
*Pseudomonas putida*	CSV86	MNAP	[125]
*Pseudomonas fluorescens*	BS3760	PHE, CHR, BaA	[50, 147]
*Pseudomonas stutzeri*	P15	PYR	[87]
*Pseudomonas saccharophilia*		PYR	[87]
*Pseudomonas aeruginosa*		PHE	[148]
*Ralstonia* sp.	SBUG 290 U2	DBF NAP	[103] [152]
*Rhodanobacter* sp.	BPC-1	BaP	[149]
*Rhodococcus* sp.		PYR, FLA	[65, 86]
*Rhodococcus* sp.	WU-K2R	NAT, BT	[121]
*Rhodococcus erythropolis*	I-19	ADBT	[120]
*Rhodococcus erythropolis*	D-1	DBT	[122]
*Staphylococcus* sp.	PN/Y	PHE	[63]
*Stenotrophomonas maltophilia*	VUN 10,010	PYR, FLA, BaP	[64, 90]
*Stenotrophomonas maltophilia*	VUN 10,003	PYR, FLA, BaA, BaP, DBA, COR	[66, 95]
*Sphingomonas yanoikuyae*	R1	PYR	[87]
*Sphingomonas yanoikuyae*	JAR02	BaP	[100]
*Sphingomonas* sp.	P2, LB126	FLE, PHE, FLA, ANT	[54, 71, 72, 150]
*Sphingomonas* sp.		DBF, DBT, CBZ	[105]
*Sphingomonas paucimobilis*	EPA505	FLA, NAP, ANT, PHE	[36, 151]
*Sphingomonas wittichii*	RW1	CDD	[112]
*Terrabacter* sp.	DBF63	DBF, CDBF, CDD, FLE	[48, 109, 117]
*Xanthamonas* sp.		PYR, BaP, CBZ	[93]

PYR, pyrene; BaP, Benzo[*a*]pyrene; PHE, phenanthrene; FLA, fluoranthene; FLE, fluorene; ANT, anthracene; NAP, naphthalene; BaA, benz[*a*]anthracene; dMBaA, dimethylbenz[*a*]anthracene; DBA, dibenz[*a,h*]anthracene; COR, coronene; CHR, chrysene; DBF, dibenzofuran; CDBF, chlorinated dibenzothophene; HFBT, 3-hydroxy-2-formylbenzothiophene; BP, biphenyl; CBP, chlorobiphenyl; NAT, naphthothiophene; BT, benzothiophene; BZ, benzoate; ADBT, alkylated dibenzothiophene; CBZ, carbazole; DD, dibenzo-*p*-dioxin; CDD, chlorinated dibenzo-*p*-dioxin; MNAP, methyl naphthalene.
